# Toward developing more effective screening questionnaires for obstructive sleep apnoea: a conceptual model and testing

**DOI:** 10.1007/s00405-025-09712-2

**Published:** 2025-10-18

**Authors:** Viktória Molnár, András Molnár, László Tamás, Jorgosz Denkler, Zoltán Lakner

**Affiliations:** 1 Department of Otorhinolaryngology and Head and Neck Surgery, Semmelweis University, Budapest, Hungary; 2 Opera Clinic, Protone Audio Kft, Lázár U. 4, 1065 Budapest, Hungary; 3 Department of Voice, Speech and Swallowing Therapy, Faculty of Health Sciences, Semmelweis University, Budapest, Hungary; 4 Szent István Campus, Hungarian University of Agriculture and Life Sciences, Gödöllő, Hungary; 5 Taskhent State Agrarian University Universitet Ko’chasi 2, Toshkent, Uzbekistan

**Keywords:** Obstructive sleep apnoea, Screening, Questionnaire, Prediction, Structural Equation modelling

## Abstract

**Purpose:**

**‘**Paper-and-pencil’ questionnaires are essential tools for the preliminary screening of obstructive sleep apnoea (OSA), a condition that is rapidly becoming more common yet often remains underdiagnosed. The most commonly used simple tests were developed several decades ago and require updating. This research aims to gain a better understanding of the various factors that contribute to the development of OSA.

**Methods:**

A total of 911 patients participated in the study and completed a self-designed questionnaire that assessed their perceptions of the intensity of various risk factors for OSA, associated comorbid conditions, sleep quality, and lifestyle-related characteristics. Following this, polysomnography was conducted to objectively evaluate the severity of OSA. The relationships among predisposing and contributing factors, as well as the perceived symptoms of OSA, were analysed using partial least squares regression.

**Results:**

Our findings emphasise the crucial roles of sex, body mass index, age, and the presence of gastroesophageal reflux disease in the development of OSA. Therefore, questions about these factors should be included in a future, updated questionnaire. The relationships between sleep quality, quality of life, and OSA is highly complex; both their values and the directions of their regression parameters vary significantly between OSA patients and those without OSA. It is possible to distinguish OSA patients with 91.87% accuracy, achieving high sensitivity (78.2%) and specificity (96.5%), based on the structure of path and regression coefficients.

**Conclusions:**

Our findings indicate opportunities for developing more effective and targeted questionnaires that remain relatively simple without being overly simplified. We discovered that a two-step question format could yield the most beneficial outcomes.

## Introduction

Obstructive sleep apnoea (OSA) is the most prevalent type of sleep-related breathing chronic disorder, affecting nearly one billion people and significantly diminishing their quality of life (QoL) [1–3]. The condition is characterised by recurrent upper airway obstruction during sleep, leading to hypoxia, hypercapnia, and increased sympathetic activity. Estimating the true burden of OSA is difficult. This challenge arises not only because OSA is associated with conditions like ischaemic stroke and myocardial infarction, which result in significant mortality and years lost due to disability, but also because OSA has been definitively linked to various types of accidents [4, 5]. Despite its characteristic symptoms, OSA is often underdiagnosed, with rates of misdiagnosis reaching as high as 80%, according to the American Academy of Sleep Medicine. [6].

The advancement of modern diagnostic methods for OSA provides reliable solutions [7]. Despite being considered the ‘gold standard’ for diagnosing OSA, the widespread use of polysomnography (PSG) incurs significant costs. These expenses are frequently not covered by social security systems, even in relatively developed countries. For instance, in Hungary, which ranks among the top 60 countries by various annual Gross Domestic Product (GDP) per capita indicators, the wait time for PSG exceeds six months. The high prevalence of OSA, combined with the costs and limited availability of PSG, has led to the increased use of screening devices. Cardiorespiratory polygraphies help alleviate the burden on sleep laboratories, reduce waiting times for examinations, and provide financial benefits. Type III portable monitoring devices are suitable for screening OSA in a home setting. [8]. While these devices offer many benefits, they yield less accurate results compared to PSG. Sensor displacement can result in insufficient data, and due to the high prevalence of OSA, even screening devices often have waiting lists of several months. Consequently, the quick and simple ‘paper-and-pencil’ methods for OSA screening are crucial tools in both general practice and clinical settings. It is clear that the performance of currently used questionnaires is relatively weak, and their practical applicability is widely debated, particularly regarding the Epworth [9, 10], Berlin [11] and STOP-BANG questionnaires [12]. Consequently, there is growing demand for the development of modern and reliable questionnaires.

The current study aims to propose an explanatory model for OSA screening and to evaluate its effectiveness within an adult population. This article presents four key novelties: (1) it introduces a conceptual model for the preliminary screening of OSA, and (2) this model is tested using various explanatory modelling methods. To the best of our knowledge, this is the first attempt to apply this approach, which utilises state-of-the-art statistical tools. We focus on (3) analysing the structure of stochastic relationships among groups of patients with no or varying levels of OSA, and (4) demonstrating the applicability of a modern structural equation modelling (SEM) tool and the partial least squares (PLS) regression method for hypothesis testing in clinical research practice.

The article is organised as follows: The first section introduces our hypothesis system, detailing the theory and practical application of building and testing conceptual models using SEM. The second section outlines the data collection process, including the patients involved, the methods used, and discusses the results. Finally, the article concludes with a summary of the key findings and provides several recommendations.

## Methodology

### Development of hypotheses and conceptual models

Based on a comprehensive analysis of the literature [13], our model-building began with four key assumptions: (1) OSA arises from various factors that may be partially or completely independent of each other; (2) many of the phenomena associated with the consequences of OSA are quite abstract [14, 15], such as ‘sleep quality’ or ‘low energy levels during the day.’ Thus, measuring it is impossible; only certain questions that are assumed to relate to this theoretical or background variable can be posed. For instance, we could inquire, ‘Do you feel tired in the morning?’ In this way, background variables can be described as a combination of responses to simple, interpretable questions measured on a binary scale (i.e., yes-or-no-type dichotomy questions). (3) OSA leads to insufficient sleep, resulting in low perceived energy levels [16], which can be assessed through self-reported morning fatigue and daily sleepiness. The combination of poor sleep quality and low energy can contribute to mental health issues, including depression and stress. Therefore, by obtaining self-reported data on sleepiness, depression, and stress, the presence and severity of OSA can be better estimated. However, it must be noted that respondents may conceal their true experiences when filling out self-report questionnaires. This is particularly true for those who may fear that admitting to daytime sleepiness could lead to losing their driving license. Therefore, this aspect must be considered in applying this information in real life. (4) Conversely, OSA can be characterised by a measurable value, namely the apnoea-hypopnoea index (AHI).

The following hypothesis has been formulated based on theoretical premises and current literature.

- H_1_ OSA can be understood through several basic demographic and anthropometric factors [17]. Therefore, it was proposed that, based on hypotheses H_1a_ (age), H_1b_ (sex), and H_1c_ (body-mass index or BMI), a background (latent) variable can be created that strongly correlates with the AHI.
- H_2_ Health issues, not solely related to OSA, can exacerbate OSA symptoms. Among these issues, we have identified two common problems:
- H_2a_ (The presence of gastroesophageal reflux or GERD [18] and the H_2b_ (nasal breathing problems [19] increases the level of the AHI. Consequently, a latent variable derived from these indicators will serve as a suitable indicator of OSA.
- H_3_ There is a positive correlation between allergic reactions in the respiratory system and OSA [20].
- H_4_ If a patient reports H_4a_ (morning tiredness) and H_4b_ (daily sleepiness,) these two directly perceivable indicators may suggest a latent variable (i.e., perceived energy level), which positively correlates with AHI [21].
- H_5_ Sleep quality can be defined by H_5a_ (the presence or absence of various sleep-related complaints [22]), such as H_5b_ (dry mouth and morning snuffles [23]), and H_5c_ (snoring [24]), as reported by the patients or their sleeping partners. This factor is closely linked to the AHI value.
- H_6_ The patients’ QoL can be measured by perceived H_6a_ (frustration), H_6b_ (depression,) and H_6c_ (stress), and H₆_d_ (QoL is directly influenced by OSA) [25]. Patients with a higher AHI often report lower QoL [26] and reduced perceived energy levels. Therefore, QoL indicators may be useful for estimating AHI.
- H_7_ The hypotheses H_1_ through H_6_ can be generalised to apply to different groups of patients.

#### Study population

The study involved a total of 911 patients (652 men and 259 women, mean age ± SD, 43.1 ± 12.22 years). The study population consisted of patients referred to the Department of Otorhinolaryngology and Head and Neck Surgery at Semmelweis University in Budapest, Hungary. They came from various healthcare institutions across Hungary and were experiencing snoring or suspected OSA. All participants provided their consent to participate in the study. Patients under 18 years of age and those who completed a sleep study with less than 4 h of recorded test time were excluded from the sample.

The data collection process consisted of three phases: (1) measuring patients’ basic anthropometric features, such as weight, height, and body mass; (2) having participants complete a questionnaire with questions supposedly relevant to diagnosing OSA; and (3) determining various breath-related parameters through PSG.

#### Anthropometric measurements

Patients’ weight (in kg) and height (in cm) were measured, and their BMI was calculated accordingly. Based on the WHO recommendations from 1995, patients were classified into three BMI groups: normal weight (BMI ≤ 25 kg/m^2^), overweight (25 kg/m^2^ < BMI < 30 kg/m^2^), and obese (BMI ≥ 30 kg/m^2^) [27].

#### Self-reported questionnaire

In the context of the current research, a key aspect was the completion of a questionnaire designed by one of the authors of this article (V.M.). This questionnaire was informed by international literature as well as her personal experiences. This questionnaire included pertinent questions about patients’ sleep quality and quantity, their current complaints, and any existing conditions. The English translation of the questionnaire can be found in Appendix 1 und the Hungarian version can be found in Appendix 2.

Creating questionnaires is a complex process that requires balancing the amount of information collected with the burden placed on respondents. We realised that using psychometrically validated scales for each variable would not be practical. For instance, we could have included additional anthropometric measurements, such as neck circumference, and certain background variables could have been assessed with more precision.

To illustrate, the well-known RAND-36 questionnaire includes four items to evaluate perceived energy levels. In our study, we utilised two questions for this assessment: one from the RAND-36 and another that we developed specifically to measure perceived energy levels in the morning. This latter variable was deemed important for estimating the impact of sleep quality on energy levels the following day.

Measuring QoL with just three questions may seem overly simplistic, especially since most standard QoL questionnaires typically assess 5 to 9 distinct dimensions [28]. However, there are two key points to consider: (1) for over four decades, it has been widely accepted that QoL can be effectively characterised by the presence or absence of frustration, stress, and depression; [29] and (2) we believe that self-assessment of these emotional states is sufficient for screening OSA, as there is a strong correlation between these mental states and sleep quality [30].

#### Sleep examination

The sleep studies were conducted at the Department of Otorhinolaryngology and Head and Neck Surgery of Semmelweis University in Budapest, Hungary. The results were evaluated according to the latest recommendations of the American Academy of Sleep Medicine [31]. In this study, apnoea was defined as a reduction in airflow of 90% or more on the oronasal thermistor signal, lasting for at least 10 s. Meanwhile, hypopnoea was defined as a reduction in airflow of 30% or more, accompanied by either a desaturation of oxygen-haemoglobin of 3% or greater, or an arousal. OSA severity was classified based on the AHI, which measures the frequency of apnoea and hypopnoea events per hour [32]. Patients with an AHI of less than 5 events per hour were placed in the control group (*n* = 450 participants). Those with an AHI of 5 events/hour ≤ AHI < 15 events/hour were classified as having mild OSA (*n* = 255 participants), while those with 15 events/hour ≤ AHI were categorised as moderately-severe to severe OSA (*n* = 206 participants). Patients with moderate and severe OSA were combined into a single category of moderately severe to severe OSA due to the small sample size.

#### Statistical analysis

Responses to the questionnaires, along with basic demographic and anthropometric data, as well as the results of PSG, were collected between 2014 and 2024 and recorded in a spreadsheet table (Microsoft 365, Excel ™).

The hypothesis testing was conducted using SEM. The main objective of SEM is to develop a framework for linear models using simultaneous regression equations that incorporate latent variables. Similar to those found in factor analysis, these are created from a combination of measurable variables. There are two types of SEMs, i.e., the covariance-based approach and the variance-based partial least squares approach. The mathematical foundations of the two models are different, yet their results are comparable [33]. Covariance-based models are primarily used to validate theories, while the partial least squares (PLS) regression method is employed for causal-predictive approaches [34]. In our calculations, the Smart_PLS™ software was used.

Given its flexibility and versatility, the latter method was utilised for both ordinary and binary data [35]. The theoretical foundation of the two methods is outlined as follows. The model includes two components: the measurement model defines how directly observed variables, such as age or feelings of depression, relate to a non-observable latent variable (or construct) like ‘demographic position’ or ‘QoL’. The structural model quantifies the hypothesised relationships among latent variables. SEMs are commonly used in psychology, sociology, and marketing; however, their application in medical sciences is relatively uncommon.

In simple terms, we developed a model to explain the relationship between OSA and several predisposing factors and symptoms. For some aspects of the model, a single variable was enough to convey the necessary information. However, in most cases, we relied on at least two directly measurable variables.

In the next phase, we applied this model to the original dataset and evaluated its fit using a variety of indicators. Based on the statistical relationships between the variables, we automatically classified patients into either the OSA group or the non-OSA group. After this classification, we re-fitted the conceptual model separately for each group. We also applied machine learning methods to classify respondents based on their questionnaire answers.

The importance–performance matrix of various directly measurable indicators related to AHI, along with the stepwise summary, is presented in Appendix 3.

**Fig. 1:**
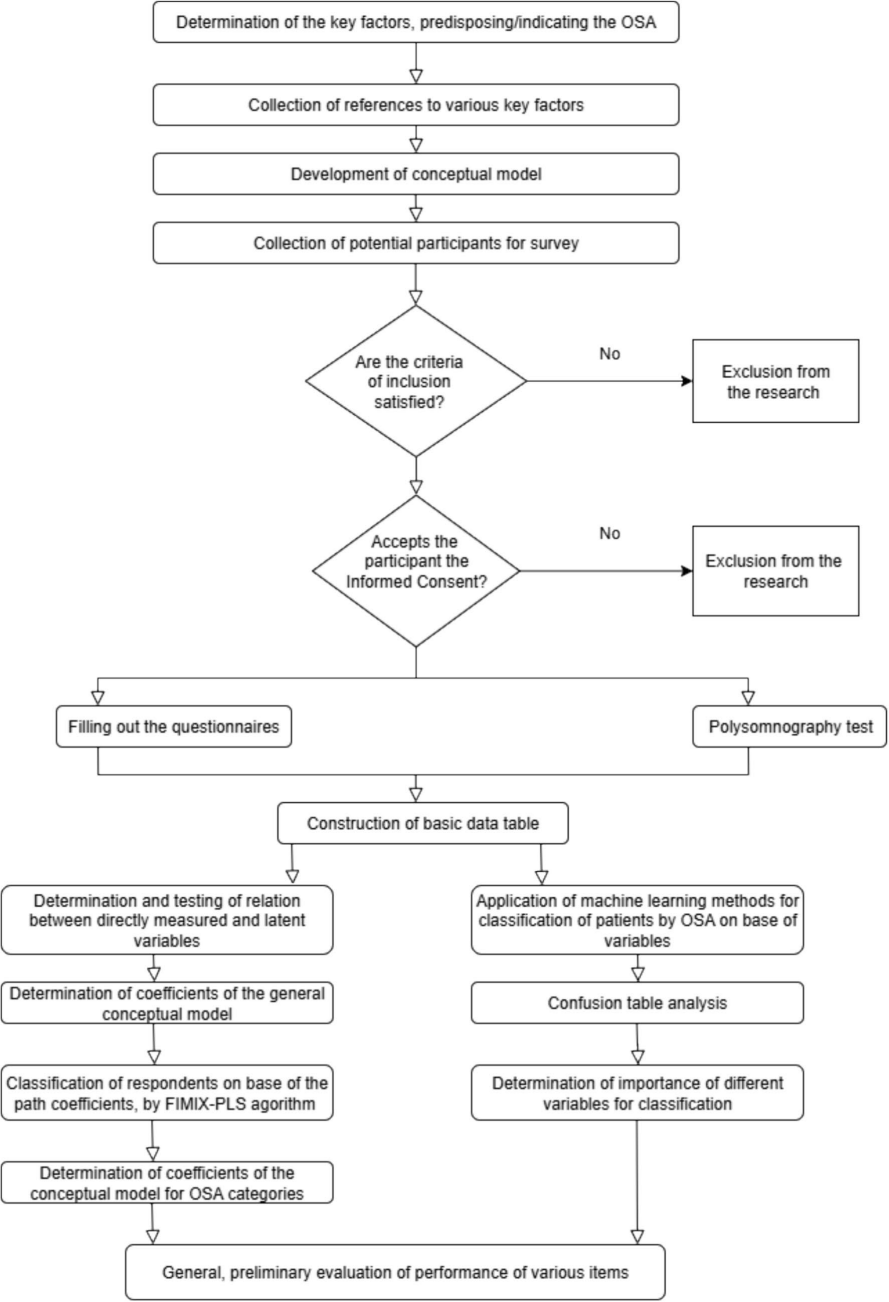
Detailed flowchart of the investigation. OSA = obstructive sleep apnoea

A detailed flowchart of the investigation is shown in Fig. 1.

## Results

The most important characteristic features of the population are summarised in Table 1.

**Table 1:** The most significant characteristics of the study sample. AHI = apnoea-hypopnoea index, BMI = body mass index, OSA = obstructive sleep apnoea

	# of patients [*n (%)*]
Sex	
Men	652 (71.5%)
Women	259 (28.5%)
Age categories	
Under 40 years	370 (40.6%)
40 years old or older	541 (59.4%)
BMI categories	
Normal (BMI ≤ 24.9 kg/m^2^)	157 (17.2%)
Overweight (24.9 kg/m^2^ < BMI ≤ 29.9 kg/m^2^)	415 (45.6%)
Obese (BMI > 29.9 kg/m^2^)	339 (37.2%)
AHI values	
Normal (AHI ≤ 5 events/hour)	450 (49.4%)
Mild OSA (5 events/hour < AHI ≤ 15 events/hour)	255 (28.0%)
Moderate and severe OSA (AHI > 15 events/hour)	206 (22.6%)

Table 2 presents the summary statistics for continuous variables in our study, including their means, standard deviations, and ranges.

**Table 2:** Descriptive statistics for continuous variables. AHI = Apnoea–Hypopnea Index; RI = Respiratory Disturbance Index; ODI = Oxygen Desaturation Index

	Mean	Median	Standard deviation	Interquartile range	Minimum	Maximum	25th percentile	75th percentile
Age (years)	43.1	43	12.2	17	18	80	34	51
Weight (kg)	89.3	88	18.1	22	35	180	78	100
Height (cm)	176	176	- 9.9	13	123	198	170	183
BMI (kg/m^2^)	28.9	28	5.5	6.5	17	58	25	31.5
AHI (events/h)	11.6	5.4	16.1	12	0	115	2	14
RI (events/h)	14.8	9	16	13	1	117	5	18
ODI (events/h)	11.8	6	14.5	12.3	0	85	2.7	15
Lowest saturation (%)	81.6	83	8.6	9	0	97	78	87
Baseline saturation (%)	95.4	96	3.8	2	0	100	95	97

The data presented in Table 1. indicates that about two-thirds of the 911 patients in the study were male. Most of these patients were over 40 years old and were classified as overweight or obese based on their BMI. The sample included half of the patients in the control group, while 25% were categorised as having mild OSA and the remaining 25% were classified as moderately severe OSA.

**Fig. 2:**
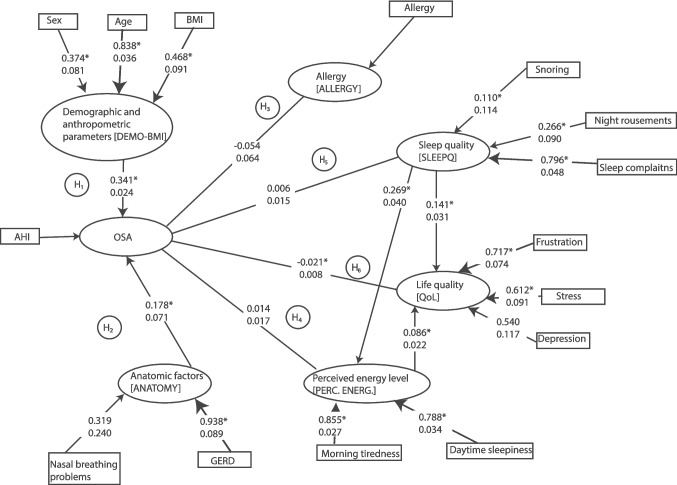
The conceptual model with hypotheses and coefficients, short names of the various background (latent) variables are indicated in square brackets. AHI = apnoea-hypopnoea index, BMI = body mass index, GERD = gastroesophageal reflux disease, H = hypothesis, OSA = obstructive sleep apnoea, PERC. ENERG. = perceived energy level, SLEEPQ = Sleep Quality. Standardised path coefficients, also known as normalised linear regression coefficients (hereinafter referred to as path coefficients), along with their standard deviations, were computed based on the structure of the path model. The significance level at *p* < 0.05 is indicated by an asterisk (*). The thickness of the arrows is roughly proportional to the strength of the correlation

A measurement model was assessed in the first phase by evaluating indicator reliability. The results of these calculations are summarised in Fig. 2.

This method involves analysing the loadings of directly measured variables. If only one variable is used to characterise a construct, its loading will be 1. The significance of the loadings was assessed using a bootstrap algorithm with 5,000 repetitions. All loadings were found to be significant, except for the variables ‘problems in nasal breathing’ and ‘snoring.’ Loadings above 0.708 are typically considered significant, as this threshold indicates that the directly measured variance accounts for at least 50% of the variance in the latent variable. If this more stringent criterion is applied, the variables ‘night-time sniffing’ and ‘dry mouth’ can be characterised by relatively low performance. Additionally, the indicators of sex and BMI demonstrated a relatively weak connection with the background variable. Stress plays a significant role in contributing to QoL, while QoL can also explain a considerable portion of the variation in stress levels.

The convergent validity was assessed using the Average Variance Extracted (AVE) indicator. All variables demonstrated significant results; however, the AVE values for DEMO-BMI, SLEEPQ, and QoL were 0.354, 0.326, and 0.405, respectively. These values indicate that these latent variables do not adequately explain the variance of their indicators.

The primary objective of SEM is to test hypotheses concerning the relationships between latent variables, as defined by the theoretical model. The fit of the model was evaluated using the standardised Root Mean Square Residual (SRMR) measure. This method is commonly utilised to evaluate the difference between the observed and model-implied correlation matrices. A well-fitting model has an SRMR value of less than 0.1 according to Hu and Bentler [36]. In our analysis, the calculated value was 0.060, as determined through bootstrap calculations. Another fitting criterion (i.e., d_ULS, based on Euclidean distance and d_G, using geodesic distance), can be employed to compare the original value with the confidence interval derived from the sampling distribution. The hypothesis posits that the two values originate from the same set. In this context, a non-significant result indicates a good model fit. Our results were below the 99% interval, which shows that this alternative test has proven the significance of the model.

In the next phase, the relationships among the latent variables were analysed. It is important to note that the PLS model employs path coefficients instead of regression coefficients. This indicates that when an independent variable changes by one standard deviation, the dependent variable will change by an amount equal to the path coefficient. Consequently, the path coefficients allow us to compare the strengths of the relationships between two variables more effectively. No significant relationship was observed between ALLERGY and OSA or between PERC. ENERG. and the OSA latent variable. The SLEEPQ-OSA relationship was significant at just 0.1 level. However, the relationships between ANATOMY-OSA and DEMO-BMI-OSA were significant. Interestingly, the connection between OSA and QoL was also significant, although it showed a negative correlation. Although this finding may seem counterintuitive, the detailed analysis will clarify this singularity.

In the relationship among the triad of sleepiness, SLEEPQ, and QoL, all connections were found to be positive and significant. Additionally, an indirect effect can be calculated from the various variables. Two significant effects were identified: a connection between SLEEPQ → SLEEPINESS → QoL (path coefficient value: 0.023, SD = 0.007) and DEMO-BMI → OSA → QoL (value of path coefficient: 0.007, SD = 0.003).

The next phase involved evaluating the structural differences among models for various patient groups. To accomplish this, the Finite Mixture Partial Least Squares (FIMIX-PLS) routine was applied. This approach seeks to identify unobserved heterogeneity in the structural model by assigning a probability of cluster membership to each record (patient) and estimating path coefficients for different clusters. It is important to emphasise that this is a non-supervised learning process; therefore, patients were automatically classified into two groups based solely on regression and path coefficients.

Based on the similarities in path coefficients, we can identify two distinct clusters of individuals. The first cluster consists of those with no or mild OSA, characterised by an AHI of less than 15 events per hour (referred to as NM-OSA). The second cluster includes individuals with moderately severe to severe OSA, which is defined as having an AHI of more than 15 events per hour (referred to as MS-OSA). The key characteristics of these two models are summarised in Table 3. The algorithm calculates the direct, indirect, and total effects of various latent factors, and the results are presented in Table 3., highlighting the total effects for both clusters.

**Table 3:** The path coefficients for the two-cluster approach. AHI = apnoea-hypopnoea index, ALLERG = allergy, DEMO-BMI = Demographic factors and BMI, ANTATOMY = Anatomic factors, QoL = quality of life, MS-OSA = moderately-severe and severe OSA, NM-OSA = no or mild OSA, PERC. ENGERG = perceived energy level, SLEEPQUAL = sleep quality

Latent variables	NM-OSA cluster	MS-OSA cluster
SLEEPQUAL → QoL	0.327	0.337
SLEEPQUAL → PERC. ENERG	0.316	0.472
AHI → SLEEPQUAL	−0.568	0.443
AHI → QoL	−0.470	0.376
AHI → PERC. ENERG	−0.678	0.534
ALLERG → SLEEPQUAL	0.191	0.065
ALLERG → AHI	−0.336	0.123
ALLERG → QoL	0.158	0.046
ALLERG → PERC. ENERG	0.228	0.066
ANTHRO → SLEEPQUAL	−0.171	0.146
DEMO-BMI → AHI	0.300	0.330
DEMO-BMI → QoL	−0.141	0.124
DEMO-BMI → SLEEPINESS	−0.204	0.176
ANATOMY → SLEEPQUAL	0.211	0.146
ANATOMY → AHI	−0.371	0.330
ANATOMY → QoL	0.175	0.124
ANATOMY → PERC. ENERG	0.252	0.176
PERC. ENERG. → QoL	0.263	0.142

Segmenting patients based on the relationships between latent variables was effective, achieving an accuracy rate of 91.87%, with a sensitivity of 78.2% and a specificity of 96.5%. These results indicate distinct differences in the SEM for patients with NM-OSA compared to those with MS-OSA. Among NM-OSA patients, only a small percentage (3.5%) was misclassified as MS-OSA. This method shows potential for effective preliminary screening without the need for costly diagnostic tools.

The model fitting for the two segments was significantly higher than that of the general population. The R-squared value for the NM-OSA segment was 0.468, while for the MS-OSA segment it was 0.347. The results clearly illustrate the relationship between OSA and QoL. QoL was assessed using direct variables on an ordinal scale, where higher values of this latent variable indicate lower QoL. It is evident that there is a positive relationship between the severity of OSA and the decline in QoL experienced by patients with this condition.

The results of various fitting tests for the proposed model, when compared to the actual data, were significant. This indicates that the model effectively describes the statistical relationships among the background variables outlined in the H_1_–H_9_ hypothesis system. However, it is evident that there is ample opportunity for further improving the model. This can be attributed to two main reasons: (1) some factors relevant to OSA have been necessarily neglected and require more advanced tools for analysis; (2) the set of patients was relatively heterogeneous. Another potential source of fitting errors could be the mis-specification of the model; however, a significant relationship exists between directly measured and latent variables. Therefore, further analysis of the proposed model is warranted.

## Discussion

Given the current conditions, it is important to recognise that the limited availability of advanced diagnostic instruments, along with the constraints of human and material resources, underscores the undeniable value of questionnaires. However, it is essential to further improve these questionnaires to enhance their sensitivity and specificity. The questionnaires currently in use are vital for general practitioners, occupational medicine specialists, and sleep laboratories. Utilising screening questionnaires enables a more rational allocation of medical resources.

Developing a new screening questionnaire is a complex and challenging task. One specific challenge is that symptoms can vary among patients with different levels of OSA. Furthermore, it is crucial to recognise that OSA can lead to a wide range of symptoms. Considering these factors, we aimed to outline potential directions for developing a new, modern questionnaire that takes into account the socio-cultural specificities of the population being studied. To achieve this, we conducted a thorough analysis of a large-scale patent survey.

Age, sex, and BMI are essential indicators for screening OSA, given its underlying mechanisms. Our results supported the H1 hypothesis: age, sex, and BMI significantly load in the background variable, which significantly correlates with AHI.

The H_2_ and H_3_ hypotheses were also fulfilled: GERD and nasal breathing issues increase AHI levels [37, 38]. These results align with findings from the existing literature. There is a well-documented circular relationship between GERD and OSA. OSA can significantly contribute to GERD due to pressure fluctuations experienced during sleep. Conversely, GERD can irritate the mucous membranes of the upper airway, potentially leading to the development of OSA. Nasal breathing limitations can significantly reduce sleep quality and increase the risk of OSA and snoring [39].

We have not established a significant correlation between allergic reactions and OSA, which may be attributed to the high prevalence of respiratory allergies in Central Europe [40].

The H_4_ hypothesis system was only partially fulfilled. On one hand, there was a strong correlation between morning tiredness and daily sleepiness—both of which are directly measurable variables—and the perceived energy level. However, a clear and significant relationship between these variables and the AHI level could not be established for the overall population. It is important to note that the diversity within the population can obscure the true relationships. By analysing these stochastic relationships separately for patients with and without OSA, we can observe a significant negative relationship between the patients’ AHI and their perceived energy levels. This conclusion is well-supported by existing literature [41] and specialists’ experiences in this field.

The low R-squared value can be attributed to the fact that OSA is a complex phenomenon, making it impossible to establish a simple equation between various factors [24].

However, it is important to note that OSA does not always accompany sleepiness, and conversely, not all complaints of sleepiness suggest OSA. This observation aligns with our findings and those of Fuglsang et al. [42]. This study was unable to establish a correlation between daytime tiredness and AHI. Similar findings were reported by Hayden et al. [43]. The study demonstrated a negative correlation between AHI, which increased due to COVID-19 disease, and levels of daytime sleepiness and fatigue.

The background variable of [SLEEP QUALITY] is characterised by factors such as snoring, night arousals, and sleep complaints. However, no significant connection between AHI and sleep quality was observed across the entire population. In contrast, a significant relationship was found within the population suffering from OSA. A possible explanation for the weak connection may be the generally low level of sleep hygiene in the Hungarian population, influenced by high consumption of stimulants [44], excessive gaming [45], and various other factors [46]. A clear and definitive connection can be established with the OSA population. Our H5 hypothesis was partially fulfilled.

The H_6_ hypothesis has been validated, showing a significant negative correlation between AHI and QoL. This finding aligns with common intuitions and emphasises the importance of AHI in enhancing citizens' daily lives. This connection was particularly evident in patients with OSA. Our results align with the existing literature [47]. The level of perceived energy has an impact on overall QoL. This finding was confirmed for the entire population, but the results for the non-OSA and OSA subgroups were inconclusive. One possible explanation for this inconclusiveness is the high standard deviation within the subgroups, indicating significant variations in the respondents’ QoL. When patients were grouped into OSA and non-OSA categories, a correlation was confirmed between the AHI and perceived QoL.

The H_7_ hypothesis could not be confirmed due to significant differences in the intensity of path coefficients. Notably, the sign of these coefficients varies between patients with OSA and those without. When examining the entire population, the relationships between the AHI, perceived energy level, QoL are negative. However, for patients with OSA, the relationships are positive. This fact underscores the significance of OSA in influencing patients’ QoL. It appears that creating a general equation system for the entire population is not feasible because the relationships among various factors differ between patients with OSA and those without it. However, these differences can be leveraged to differentiate between OSA patients and non-OSA patients. Our model was able to successfully classify 91.87% of the patients based on latent variables and their interconnections.

## Limitations

The current study focused on the Hungarian population, which means that its conclusions cannot be generalised to other groups. Additionally, a methodological challenge is ensuring that participants’ answers are not manipulated and that there is no bias stemming from personal interests, such as concerns about losing their driving licenses.

A more detailed analysis could have been performed by categorising the patients into three groups: no OSA, mild OSA, and severe OSA. However, we chose to use a binary classification approach for two main reasons: (1) our objective was to develop a preliminary screening tool rather than a diagnostic questionnaire, and (2) most existing machine learning methods are optimised for binary classifications. In binary classification, the confusion matrix consists of only four cells, while using three classes would expand this to nine cells, significantly increasing the potential for misclassification.

One notable limitation of our study is the significant variability in sleep patterns from night to night. This variability presents a major challenge to the accuracy of predictive models commonly used in sleep medicine. As observed in other sleep studies [48], this unpredictability introduced considerable 'noise' into our data. Therefore, conducting a longer and more comprehensive longitudinal study would be beneficial.

In future phases, further anthropometric parameters, including neck circumference, should be added to the investigations.

We must also recognise that our findings cannot be generalised, as this study was conducted in Hungary, reflecting the socio-cultural and anthropological specificities of this relatively small East-Central European country.

## Conclusions

Using a relatively large sample of adult patients, we developed and tested a conceptual model that explores the interplay of various factors related to OSA, considering both objective and subjective parameters. Based on this, suggestions can be made for further development of OSA-related screening questionnaires. The key suggestions are listed below:

1. The screening questionnaire must include questions about biological sex, age, and BMI, as these factors are significant predictors of OSA.
2. The first item in the questionnaire addressed the presence of GERD. However, issues with nasal breathing may not provide an accurate assessment of OSA. Thus, this question could be removed if a shorter questionnaire is desired. The same reasoning applies to questions related to patients’ allergies. The presence of snoring, nighttime awakenings, and sleep complaints are significant predictors of sleep quality. These factors operate in a circular relationship with OSA, which is notably significant and positive in patients suffering from OSA.
3. The connection between the AHI and the SLEEPQ-SLEEPINESS-QoL triad is quite complex. For non-OSA patients, understanding these latent variables does not offer significant benefits for categorisation. However, for OSA patients, this information is crucial. A possible strategy involves creating a two-step questionnaire. If a patient scores below a certain threshold on one of the factors outlined in the initial points, the second part of the questionnaire—focused on QoL—could provide important indicators for more accurately classifying patients based on their OSA severity.
4. Our research indicates that a questionnaire with about 20 items may effectively prescreen for OSA.
5. It is essential to consider that patients’ responses are significantly influenced by their personality types. For instance, individuals with certain personality traits, such as phlegmatic types, may focus more on their illness symptoms. In many cases, individuals may be consciously or, more frequently, unconsciously unaware of their condition and symptoms. This ‘I do not care’ attitude can greatly distort their responses. Therefore, when developing a new screening questionnaire, it is essential to consider different personality types.

In summary, OSA screening can be a vital tool for the future, particularly through the use of questionnaires. However, our results indicate that these questionnaires require updates. We recommend implementing a two-stage system of questions as an efficient approach for screening.

## Figures and Tables

**Fig. 3:**
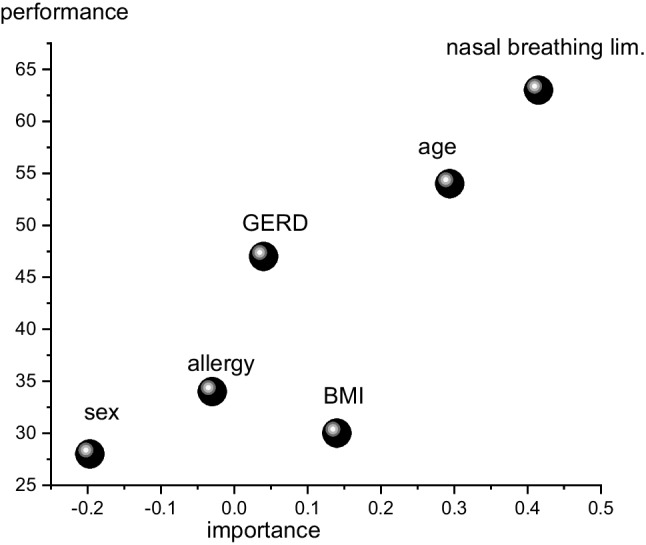
Results of the importance-performance analysis conducted using Smart_PLS. BMI = body mass index, GERD = gastroesophageal reflux disease, lim. = limitation

## Data Availability

Data will be made available on reasonable request.

## Appendix 1

Screening questionnaire for OSA.

Name:

Identification number:

Sex:

Date of birth:

1. Weight: cm
2. Height: kg
3. Difficult nose breathing? yes/no
4. Snoring? yes/no
5. Average sleep time in hours:
6. Beginning of sleep time:
7. End of the sleep time
8. Having trouble falling asleep? yes/no
9. Number of wake-ups?
10. Sleeping difficulty? yes/no
11. Nasal congestion at night, dry mouth in the morning? yes/no
12. Feeling tired in the morning? yes/no
13. Daytime sleepiness? yes/no
14. Psychological instability (e.g., anxiety, depression or stress)? yes/no. If ‘yes,’ please specify which one.
15. Do you have any allergies? yes/no. If yes, please specify what you are allergic to.
16. Gastroesophageal reflux disease? yes/no
17. Smoking? yes/no
18. Alcohol consumption? yes/no
19. Drug consumption? yes/no
20. Do you take any medications regularly? Yes or No. If yes, please list which ones.
21. Any infectious disease? yes/no
22. Any other symptoms?

## Appendix 3

The results of the analysis regarding the relative importance of various directly measured indicators.

In the first phase, the importance-performance matrix of various directly measurable indicators in relation to AHI was determined.

The analysis results (Fig. 3) are not conclusive, but these preliminary data underscore the significance of collecting information on nasal breathing limitations and age.

In the next phase of our research, the role of various indicators by a simple machine learning technique known as binary logistic modelling was analysed. Our results indicate that age, BMI, sex, snoring, and GERD are significant factors in predicting the AHI. However, it is evident that more comprehensive questionnaires are necessary to obtain more reliable results.

## Stepwise summary

BMI = body mass index, GERD = gastroesophageal reflux disease, AIC = Akaike Information Criterion, BIC = Bayesian Information Criterion.

**Table:**

Variable	Method	AIC	BIC	Deviance	
Age cat	addition	1182.890	1192.519	1178.890	
BMI cat	addition	1117.449	1131.893	1111.449	
Sex addition	1097.362	1116.621	1089.362		
Snoring addition	1079.080	1103.153	1069.080		
GERD	addition	1076.307	1105.195	1064.307	
Daytime					
Sleepiness addition	1075.335	1109.037	1061.335		
Maximum Likelihood Estimates					
Parameter	DF	Estimate	Std. Error	z value	Pr(>|z|)
(Intercept) 1	-1.9850	0.4149	-4.7839	0.0000	
Age cat	1	1.3261	0.1573	8.4332	0.0000
BMI cat	1	0.7733	0.1047	7.3824	0.0000
Sex	1	-0.8484	0.1744	-4.8638	0.0000
Snoring	1	1.3999	0.3427	4.0854	0.0000
GERD	1	0.3360	0.1680	2.0002	0.0455
Daytime					
sleepiness	1	0.2652	0.1541	1.7209	0.0853
Association of Predicted Probabilities and Observed Responses					
% Concordant	0.7400	Somers' D	0.5238		
% Discordant	0.2313	Gamma	0.5087		
% Tied	0.0287	Tau-a	0.2546		
Pairs	207450	c	0.7544		
Model Fit Statistics					
Log-Lik Intercept Only:	-631.391	Log-Lik Full Model:	-530.668		
Deviance(904):	1061.335	LR(6):	201.446		
Prob > LR:	0.000				
MCFadden's R2	0.160	McFadden's Adj R2:	0.148		
ML (Cox-Snell) R2:	0.198	Cragg-Uhler(Nagelkerke) R2:	0.265		
McKelvey & Zavoina's R2:	0.274	Efron's R2:	0.199		
Count R2:	0.683	Adj Count R2:	0.358		
BIC:	1109.037	AIC:	1075.335		
	Reference				
Prediction	0	1			
	0 297 136				
	1 153 325				
	Accuracy:	0.6828			
No Information Rate:	0.4940				
	Kappa:	0.3652			
McNemars's Test P-Value:	0.3466				
Sensitivity:	0.7050				
Specificity:	0.6600				
Pos Pred Value:	0.6799				
Neg Pred Value:	0.6859				
Prevalence:	0.5060				
Detection Rate:	0.3568				
Detection Prevalence:	0.5247				
Balanced Accuracy:	0.6825				
Precision:	0.6799				
Recall:	0.7050				

## References

[1] Grote L (2019) The global burden of sleep apnoea. Lancet Respir Med 7:645–647. 10.1016/S2213-2600(19)30226-7 31300335 10.1016/S2213-2600(19)30226-7

[2] Lyons MM, Bhatt NY, Pack AI, Magalang UJ (2020) Global burden of sleep-disordered breathing and its implications. Respirology 25:690–702. 10.1111/resp.13838 32436658 10.1111/resp.13838

[3] Borsoi L, Armeni P, Donin G, Costa F, Ferini-Strambi L (2022) The invisible costs of obstructive sleep apnea (OSA): systematic review and cost-of-illness analysis. PLoS ONE 17(5):e0268677. 10.1371/journal.pone.0268677 35594257 10.1371/journal.pone.0268677 PMC9122203

[4] Benjafield AV, Ayas NT, Eastwood PR et al (2019) Estimation of the global prevalence and burden of obstructive sleep apnoea: a literature-based analysis. Lancet Respir Med 7:687–698. 10.1016/S2213-2600(19)30198-5 31300334 10.1016/S2213-2600(19)30198-5 PMC7007763

[5] Alakörkkö I, Törmälehto S, Leppänen T, McNicholas WT, Arnardottir ES, Sund R (2023) The economic cost of obstructive sleep apnea: a systematic review. Sleep Med Rev 72:101854. 10.1016/j.smrv.2023.101854 37939650 10.1016/j.smrv.2023.101854

[6] Gurubhagavatula I et al (2023) Obstructive sleep apnea indicator report. In: T.D.S. WORKGROUP (ed) American Academy of Sleep Medicine. USA, IL

[7] Jambhekar S, Carroll JL (2008) Diagnosis of pediatric obstructive sleep disordered breathing: beyond the gold standard. Expert Rev Respir Med 2:791–809. 10.1586/17476348.2.6.791 20477240 10.1586/17476348.2.6.791

[8] Delesie M, Knaepen L, Verbraecken J, Weytjens K, Dendale P, Heidbuchel H, Desteghe L (2021) Cardiorespiratory polygraphy for detection of obstructive sleep apnea in patients with atrial fibrillation. Front Cardiovasc Med 8:758548. 10.3389/fcvm.2021.758548 34917663 10.3389/fcvm.2021.758548 PMC8669303

[9] Omobomi O, Quan SF (2018) A requiem for the clinical use of the Epworth sleepiness scale. J Clin Sleep Med 14:711–712. 10.5664/jcsm.7086 29734996 10.5664/jcsm.7086 PMC5940419

[10] Godoy PH, dos Santos Nucera APC, de Paiva CA (2022) de-Andrade JE, Alves DdSB 2022 Screening for obstructive sleep apnea in elderly: performance of the Berlin and STOP-Bang questionnaires and the Epworth sleepiness scale using polysomnography as gold standard. Sleep Sci 15:203–208. 10.5935/1984-0063.20220033 35755908 10.5935/1984-0063.20220033 PMC9210560

[11] Ahmadi N, Chung SA, Gibbs A, Shapiro CM (2008) The berlin questionnaire for sleep apnea in a sleep clinic population: relationship to polysomnographic measurement of respiratory disturbance. Sleep Breath 12:39–45. 10.1007/s11325-007-0125-y 17684781 10.1007/s11325-007-0125-y

[12] Tan A, Yin JD, Tan LW, van Dam RM, Cheung YY, Lee C-H (2016) Predicting obstructive sleep apnea using the STOP-Bang questionnaire in the general population. Sleep Med 27:66–71. 10.1016/j.sleep.2016.06.034 27938922 10.1016/j.sleep.2016.06.034

[13] Osman AM, Carter SG, Carberry JC, Eckert DJ (2018) Obstructive sleep apnea: current perspectives. Nat Sci Sleep. 10.2147/NSS.S124657 29416383 10.2147/NSS.S124657 PMC5789079

[14] Muthén BO (2002) Beyond SEM: general latent variable modeling. Behaviormetrika 29:81–117. 10.2333/bhmk.29.81

[15] Sarstedt M, Radomir L, Moisescu OI, Ringle CM (2022) Latent class analysis in PLS-SEM: a review and recommendations for future applications. J Busin Res 138:398–407. 10.1016/j.jbusres.2021.08.051

[16] Leger D, Stepnowsky C (2020) The economic and societal burden of excessive daytime sleepiness in patients with obstructive sleep apnea. Sleep Med Rev 51:101275. 10.1016/j.smrv.2020.101275 32169792 10.1016/j.smrv.2020.101275

[17] Senaratna CV et al (2017) Prevalence of obstructive sleep apnea in the general population: a systematic review. Sleep Med Rev 34:70–81 27568340 10.1016/j.smrv.2016.07.002

[18] Althoff MD et al (2021) Asthma and three colinear comorbidities: obesity, OSA, and GERD. The Journal of Allergy and Clinical Immunology: In Practice 9:3877–3884 34506967 10.1016/j.jaip.2021.09.003 PMC8578370

[19] Tsai M-S et al (2022) Holistic care for obstructive sleep apnea (OSA) with an emphasis on restoring nasal breathing: a review and perspective. J Chin Med Assoc 85(6):672–678 35507064 10.1097/JCMA.0000000000000737 PMC12755432

[20] Kimple AJ, Ishman SL (2013) Allergy and sleep-disordered breathing. Curr Opin Otolaryngol Head Neck Surg 21:277–281 23619424 10.1097/MOO.0b013e32835ff132

[21] Gonsalves MA et al (2004) Obstructive sleep apnea syndrome, sleepiness, and quality of life. Chest 125:2091–2096 15189926 10.1378/chest.125.6.2091

[22] Frangopoulos F et al (2021) The complex interaction between the major sleep symptoms, the severity of obstructive sleep apnea, and sleep quality. Front Psychiatry 12:630162 33716827 10.3389/fpsyt.2021.630162 PMC7947685

[23] Zhang C, Shen Y, Liping F, Ma J, Wang GF (2021) The role of dry mouth in screening sleep apnea. Postgrad Med J 97:294–298. 10.1136/postgradmedj-2020-137619 32913036 10.1136/postgradmedj-2020-137619

[24] Hsu Y-C (2022) Integrating domain knowledge with machine learning to detect obstructive sleep apnea: snore as a significant bio-feature. J Sleep Res 31:e13487 34549473 10.1111/jsr.13487

[25] Kuhn E et al (2017) Effects of CPAP and mandibular advancement devices on health-related quality of life in OSA: a systematic review and meta-analysis. Chest 151:786–794 28130044 10.1016/j.chest.2017.01.020

[26] Abma IL, van der Wees PJ, Veer V, Westert GP, Rovers M (2016) Measurement properties of patient-reported outcome measures (PROMs) in adults with obstructive sleep apnea (OSA): a systematic review. Sleep Med Rev 28:18–31. 10.1016/j.smrv.2015.07.006 26433776 10.1016/j.smrv.2015.07.006

[27] Committee WE (1995) Physical status: the use and interpretation of anthropometry. World Health Organ Tech Rep Ser 854:312–344 8594834

[28] van Krugten FCW et al (2021) Instruments to assess quality of life in people with mental health problems: a systematic review and dimension analysis of generic, domain- and disease-specific instruments. Health Qual Life Outcomes 19:249 34727928 10.1186/s12955-021-01883-w PMC8561965

[29] Abbey A, Andrews FM (1985) Modeling the psychological determinants of life quality. Soc Indic Res 16:1–34

[30] Li Y (2019) Relationship between stressful life events and sleep quality: rumination as a mediator and resilience as a moderator. Front Psychiatry 10:348 31191370 10.3389/fpsyt.2019.00348 PMC6545794

[31] Kapur VK, Auckley DH, Chowdhuri S, Kuhlmann DC, Mehra R, Ramar K, Harrod CG (2017) Clinical practice guideline for diagnostic testing for adult obstructive sleep apnea: an American Academy of Sleep Medicine clinical practice guideline. J Clin Sleep Med 13:479–504. 10.5664/jcsm.6506 28162150 10.5664/jcsm.6506 PMC5337595

[32] Malhotra A, Ayappa I, Ayas N, Collop N, Kirsch D, Mcardle N, Mehra R, Pack AI, Punjabi N, White DP, Gottlieb DJ (2021) Metrics of sleep apnea severity: beyond the apnea-hypopnea index. Sleep 44:zsab030. 10.1093/sleep/zsab030 33693939 10.1093/sleep/zsab030 PMC8271129

[33] Hair JF, Ringle CM, Sarstedt M (2011) PLS-SEM: indeed a silver bullet. J Market Theory Pract 19:139–152. 10.2753/MTP1069-6679190202

[34] Hair Jr JF, Hult GTM, Ringle CM et al (2021) An introduction to structural equation modeling. In: Partial least squares structural equation modeling (PLS-SEM) using R: a workbook. pp 1–29

[35] Hair Jr JF, Matthews LM, Matthews RL, Sarstedt M (2017) PLS-SEM or CB-SEM: updated guidelines on which method to use. Int J MultivarData Anal 1:107–12310.1504/IJMDA.2017.087624

[36] Hu L, Bentler PM (1998) Fit indices in covariance structure modeling: sensitivity to under parameterized model misspecification. Psychol Methods 3:424–453. 10.1037/1082-989X.3.4.424

[37] Mahfouz R, Barchuk A, Obeidat AE, Mansour MM, Hernandez D, Darweesh M, Aldiabat M, Al-Khateeb MH, Yusuf MH, Aljabiri Y (2022) The relationship between obstructive sleep apnea (OSA) and gastroesophageal reflux disease (GERD) in inpatient settings: a nationwide study. Cureus 14(3):e22810. 10.7759/cureus.22810 35399477 10.7759/cureus.22810 PMC8980249

[38] Tsai M-S, Chen H-C, Liu SY-C et al (2022) Holistic care for obstructive sleep apnea (OSA) with an emphasis on restoring nasal breathing: a review and perspective. J Chin Med Assoc 85:672–678. 10.1097/JCMA.0000000000000737 35507064 10.1097/JCMA.0000000000000737 PMC12755432

[39] Cai Y, Goldberg AN, Chang JL (2020) The nose and nasal breathing in sleep apnea. Otolaryngol Clin North Am 53(3):385–395. 10.1016/j.otc.2020.02.002 32192710 10.1016/j.otc.2020.02.002

[40] Leru PM, Anton VF, Chovancova Z et al (2024) Evaluation of respiratory allergies burden and management in primary care and comparative analysis of health care data from Romania, Poland, Czech Republic and Bulgaria–preliminary study. Rom J Intern Med 62(3):341–355. 10.2478/rjim-2024-0018 38656830 10.2478/rjim-2024-0018

[41] Gottlieb DJ (2022) Screening for obstructive sleep apnea in adults. JAMA 328:1908–1910. 10.1001/jama.2022.20670 36378222 10.1001/jama.2022.20670

[42] Fuglsang M, Lilja-Fischer JK, Petersen KB, Bille J (2019) Subjective tiredness does not correlate with the Apnoea-Hypopnoea index. Dan Med J 66:A5545 31066355

[43] Hayden MC, Schwarzl G, Limbach M, Mitrea S, Schuler M, Nowak D, Schultz K (2022) Negative association between fatigue and signs of sleep apnoea in patients after COVID-19. ERJ Open Res 8:00289. 10.1183/23120541.00289-2022 36575709 10.1183/23120541.00289-2022 PMC9510902

[44] Sejbuk M, Mirończuk-Chodakowska I, Witkowska AM (2022) Sleep quality: a narrative review on nutrition, stimulants, and physical activity as important factors. Nutrients 14:1912. 10.3390/nu14091912 35565879 10.3390/nu14091912 PMC9103473

[45] Kristensen JH, Pallesen S, King DL, Hysing M, Erevik EK (2021) Problematic gaming and sleep: a systematic review and meta-analysis. Front Psychiatry 12:675237. 10.3389/fpsyt.2021.675237 34163386 10.3389/fpsyt.2021.675237 PMC8216490

[46] Wang F, Bíró É (2021) Determinants of sleep quality in college students: a literature review. Explore 17:170–177. 10.1016/j.explore.2020.11.003 33246805 10.1016/j.explore.2020.11.003

[47] Vogler K, Daboul A, Obst A (2023) Quality of life in patients with obstructive sleep apnea: results from the study of health in Pomerania. J Sleep Res 32(1):e13702. 10.1111/jsr.13702 36053870 10.1111/jsr.13702

[48] Tschopp S et al (2021) Night-to-night variability in obstructive sleep apnea using peripheral arterial tonometry: a case for multiple night testing. J Clin Sleep Med 17:1751–1758 33783347 10.5664/jcsm.9300 PMC8636340

